# Causes and Incidence of Miniplate Removal Following Le Fort I Osteotomy

**Published:** 2009-10-12

**Authors:** Afshin Haraji, Mohammad Hosein Kalantar Motamedi, Nima Moharamnejad

**Affiliations:** ^a^ Department of Oral and Maxillofacial Surgery, Azad University of Medical Sciences, Tehran, Iran; ^b^ Trauma Research Center, Baqiyatallah University of Medical Sciences, Tehran, Iran; ^c^ Private practice, Tehran, Iran

## Abstract

**Aim:** This study assessed the causes and incidence of miniplate removal during a 5-year period after Le Fort I osteotomy. **Patients and methods:** One hundred forty-two patients had plates inserted for fixation of the maxilla after Le Fort I osteotomy between 2001 and 2004. The Le Fort I segment was rigidly fixed with four 2-mm titanium miniplates and 16 screws. They were followed 1 to 5 years for plate complications and need for plate removal. **Results:** Fifteen of 142 patients (10.6%), 9 females and 6 males, required plate removal. The minimum time period between insertion and removal was 4 months and the maximum period was 18 months. Causes for removal were as follows: infection (40%), pain (13.3%); sinusitis (13.3%); sensitivity to temperature change (13.3%); palpability of plate (13.3%); and phobia (6.8%). **Conclusion:** In this study, the number of miniplates removed was small and required removal no sooner than 4 months postoperatively (after complete bony union), thus not compromising healing. There is no evidence from this study to support the routine removal of titanium miniplate after Le Fort I osteotomy, rather they should be removed when indicated.

Use of rigid fixation in oral and maxillofacial surgery has become a routine for the last 3 decades.^1^ Titanium miniplates facilitate orthognathic, craniofacial, and reconstruction surgeries. The benefits of using rigid fixation are stability of the osteotomized segment, obviating the need for intermaxillary fixation, patient comfort, and less possibility of complications in comparison with maxillomandibular fixation (such as aspiration and respiratory distress). However, there exists much controversy with regard to plate removal after healing. Champy et al^1^ suggested that plates be removed 3 months after insertion. Many authors do not support this suggestion.^2^–^6^ Allowing rigid fixation to remain may pose disadvantages such as infection, temperature sensitivity, sinusitis, palpability (by patients), and nerve and dental injuries.^6^,^7^ In 1987, Beals and Munro^8^ showed that only 1% of 74 patients who underwent craniofacial surgery required plate removal. In 1992, Francel et al^9^ reported infection in 12% of facial trauma patients who had jaw reconstruction mandating plates and screws to be removed. Exposure of the plate into the oral cavity is a common cause of removal. The aim of this study was to assess the causes and incidence of miniplate removal during a 5-year period after Le Fort I osteotomy.

## MATERIALS AND METHODS

One hundred forty-two patients had plates inserted for fixation of the maxilla after Le Fort I osteotomy between 2001 and 2004. Every patient had surgery for similar deformities, with 2 miniplates placed bilaterally (L-form with 4-hole 2-mm diameter miniplates and 7-mm screws were used). The Le Fort I segment was rigidly fixed with four 2-mm titanium miniplates and 16 screws.

All plates were inserted in the zygomatic buttress and piriform rim. Age, sex distribution, incidence, and causes for plate removal were recorded during this period. They were followed 1 to 5 years for plate complications and indications for plate removal.

## RESULTS

Fifteen of 142 patients (10.6%), 9 females and 6 males, required plate removal. The minimum time period between insertion and removal was 4 months and the maximum period was 18 months. Causes for removal were infection (40%), pain (13.3%), sinusitis (13.3%), sensitivity to temperature change (13.3%), palpability of plate (13.3%), and phobia (6.8%).

**Figure 1:**
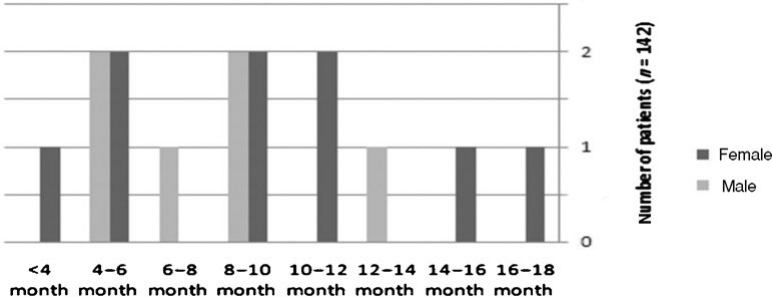
Time interval between plate insertion and removal.

The mean age of patients was 21 years (range = 18–32 years). In 15 patients (10.6%), 1 or more plates were removed. Of these patients who had plates removed, 9 (60%) were female (mean age = 24 years) and 6 (40%) were male (mean age = 25 years). Time interval between plate insertion and removal was 13.5 months (ranging from 4 to 18 month; Fig 1).

**Figure 2:**
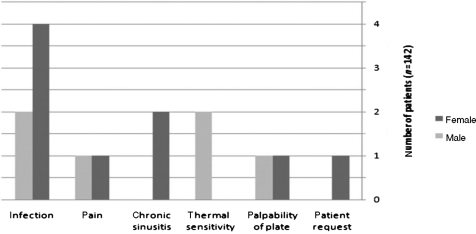
Reasons for plate removal.

The major causes for removal were as follows: infection due to wound dehiscence's and exposure of the plate in the oral cavity (6 patients, 40%), pain (2 patients, 13.3%), sinusitis (2 patients, 13.3%), sensitivity to temperature change (2 patients, 13.3%), palpability of the plate (2 patients, 13.3%), and patient phobia in asymptomatic patients (1 patient, 6.8%), as shown in Figure 2.

## DISCUSSION

During the last 2 decades, plates and screws for rigid fixation in Le Fort I osteotomy has become routine in the treatment of dentofacial deformities worldwide. Rigid fixation with plates and screws, however, is not without complications (infection, pain, dehiscence, etc). The controversy regarding retention of asymptomatic bone plates used for orthognathic surgery is ongoing. Many centers^1^,^10^,^11^ have advocated the routine removal of miniplates, whereas in other centers,^12^,^13^ they are routinely left in place. This study indicated several causes for plate removal, such as infection, plate loosening, palpability, temperature sensitivity, sinusitis, pain, and patient request (without clinical symptoms). We opted to focus only on Le Fort I osteotomy patients because the maxilla is immobile.

In this study, the patients were mostly young (mean age = 21 years) and healthy women (63%). In the current study, 10.6% of plates inserted were removed. Several article analyzed the rates of plate removal in a study made up of both orthognathic and trauma patients, and a few analyzed the rates of plate removal in orthognathic surgery, especially Le Fort I osteotomy alone. Bruzual^14^ in a study reported a lower plate removal rate (7%). In another study, Manor and Chaush^15^ found that 12% of plates inserted were removed.

The postoperative time of plate removal is controversial in the literature. In this series, the average interval between plate insertion and removal was 13.5 months. According to Mosbah et al,^12^ most plates were removed in trauma patients within 6 months following treatment, whereas Francel et al^9^ and Bakathir et al^16^ founded this rate was 9.4 and 11.5 months, respectively. In our study, time interval between plate insertion and removal was longer than the other studies; this may be because trauma patients have various types and severity of injury, soft-tissue coverage, contamination, etc.

In this study, infection was the major cause of plate removal (40%). Bhatt et al^13^ and Rallis et al^17^ founded that the chief cause for plate removal was infection (50% and 46%). This low infection rate was similar to our study.

In Le Fort I osteotomy, continuity of sinus mucosa is disturbed, the sinus is entered, and possibility of maxillary sinusitis is increased. Kahnberg and Engstrom^7^ demonstrated that in 44 patients who had Le Fort I osteotomies with wire osteosynthesis, 90% of patients had sinus mucositis after surgery. After 6 month, 50% of patients showed bilateral sinus inflammation. In our study, only 2 patients had continual sinusitis and, for this reason, fixation hardware had to be removed. After removal, sinusitis resolved.

Thermal sensitivity was another complication that was reported. Thermal sensitivity commonly accompanied pain. Alpert and Seligson^10^ showed that thermal sensitivity was a seasonal complication and had unknown cause. In this study, 2 patients presented with this problem and both of them were cured after plate removal.

Several authors reported vague pain after surgery. Mosbah et al^12^ reported the rate of pain of 14%, whereas Bhatt et al^13^ reported pain in 24%. Our result showed pain to be the cause of plate removal in 2 patients (13.3%).

Palpable plates are among one of the reasons for plate removal; in this study, 2 patients (13.3%) had plates removed for this reason. Rallis et al^17^ reported 19% of plates removal for this reason, whereas Bhatt et al^13^ reported an 11% rate.

## CONCLUSION

On the basis of this study, the number of miniplates removed was small and required removal no sooner than 4 months postoperatively (after complete bony union), thus not compromising healing. There is no evidence from this study to support the routine removal of titanium miniplate after Le Fort I osteotomy, rather they should be removed when indicated.
